# Cognitive screening and education: assessing the Montreal Cognitive Assessment’s validity in older Ugandan populations

**DOI:** 10.20935/mhealthwellb7804

**Published:** 2025-07-18

**Authors:** Kamada Lwere, Haruna Muwonge, Hakim Sendagire, Joy Louise Gumikiriza-Onoria, Rheem Nakimbugwe, Denis Buwembo, Noeline Nakasujja, Mark Kaddumukasa

**Affiliations:** 1 Department of Microbiology, School of Biomedical Sciences, College of Health Sciences, Makerere University, Kampala, Uganda.; 2 Department of Microbiology, Faculty of Health Sciences, Soroti University, Soroti, Uganda.; 3 Faculty of Health Sciences, Habib Medical School, Islamic University in Uganda, Kampala, Uganda.; 4 Department of Physiology, School of Biomedical Sciences, College of Health Sciences, Makerere University, Kampala, Uganda.; 5 Department of Psychiatry, School of Medicine, College of Health Sciences, Makerere University, Kampala, Uganda.; 6 Department of Epidemiology and Biostatistics, School of Public Health, College of Health Sciences, Makerere University, Kampala, Uganda.; 7 Department of Medicine, School of Medicine, College of Health Sciences, Makerere University, Kampala, Uganda.

**Keywords:** Montreal Cognitive Assessment (MoCA), cognitive screening, education, aging, sex disparities, Uganda, low- and middle-income countries (LMICs)

## Abstract

**Background:**

Cognitive screening tools such as the Montreal Cognitive Assessment (MoCA) are widely used to detect cognitive impairments. However, their accuracy in low- and middle-income countries (LMICs) may be affected by variations in educational levels. This study examined the impact of educational attainment on MoCA performance in older Ugandan adults, considering sex- and age-related differences.

**Methods:**

A cross-sectional study was conducted in Wakiso District, Uganda, involving adults aged ≥ 65 years. Their MoCA scores were analyzed in relation to their educational attainment, sex, and age. Multiple linear regression models were used to determine the independent effect of education on cognitive performance after adjusting for age and sex. Sensitivity analyses were conducted using multiple imputations for missing data.

**Results:**

Higher educational attainment was significantly associated with a better MoCA performance (β = 1.73, 95% CI: 1.22–2.24, *p* < 0.001). Age was negatively associated with MoCA scores (β = −0.13, 95% CI: −0.19 to −0.07, *p* < 0.001), whereas male sex was positively associated (β = 1.89, 95% CI: 0.56–3.22, *p* = 0.005). The interaction terms (education × sex and education × age) were not significant, indicating that the effect of education was consistent across demographic subgroups. The final regression model explained 42.7% of the variance in the MoCA scores (adjusted R^2^ = 0.43, *p* < 0.001). The sensitivity analysis confirmed the robustness of the findings.

**Conclusions:**

Educational attainment impacts MoCA performance in older Ugandans, highlighting the need for region-specific norms and culturally adapted cognitive screening in LMICs.

## 1. Introduction

Cognitive disorders, including dementia and mild cognitive impairment (MCI), are significant public health concerns, especially in low- and middle-income countries (LMICs), where an increasing life expectancy has led to an increasing disease burden [1]. By 2050, an estimated 152 million people globally will have dementia, with nearly 70% of cases in LMICs [2]. Dementia’s prevalence in sub-Saharan Africa is estimated between 2.3% and 5.9% among older adults but may be underreported due to limited healthcare infrastructure and low diagnostic rates [3]. The economic burden of dementia is growing, disproportionately impacting LMICs with inadequate formal support systems.

Addressing these challenges requires culturally appropriate cognitive screening tools and early diagnostic interventions for educationally diverse populations in low-resource settings. Early detection is crucial; however, LMICs face barriers such as limited access to trained personnel and suitable screening tools. The Montreal Cognitive Assessment (MoCA) is sensitive in detecting early cognitive decline but is influenced by sociodemographic factors, particularly education, potentially leading to misclassification in individuals with little or no formal schooling [4, 5].

The developers of the MoCA introduced a +1-point adjustment for those with 12 or fewer years of education [6] based on studies from high-income countries (HICs). However, the literacy rates, education systems, and familiarity with cognitive tests differ significantly in low- and middle-income countries (LMICs) like Uganda, where many older adults have little to no formal education [7, 8]. This raises doubts about the adequacy of the single-point adjustment in accounting for performance differences [9]. Individuals with low literacy often struggle with MoCA tasks, potentially leading to overestimated cognitive deficits in populations with limited schooling [10, 11].

Several LMICs have adapted the MoCA scoring for educational variability. In India, a cutoff of 19/30 was suggested for those with less than five years of education [12]. Brazil’s MoCA-BR includes simplified items and stratified scores [13]. China introduced the MoCA-B for low-literacy rural populations and found that the standard +1 correction was inadequate [14]. These adaptations indicate that a uniform adjustment to the MoCA is unsuitable across all LMICs, though a consensus on the adjustments, especially in sub-Saharan Africa, remains lacking [15, 16].

Although the MoCA is widely used, other cognitive screening tools have been developed to improve the diagnostic accuracy in low-literacy populations. The Rowland Universal Dementia Assessment Scale (RUDAS) minimizes linguistic and literacy dependence, making it more culturally fair [17]. The Instrumental Activities of Daily Living for the Elderly Assessment (IDEA) cognitive screen, validated in some LMIC settings, offers a simpler tool for dementia detection [18], while Addenbrooke’s Cognitive Examination (ACE-III) has been adapted for cross-cultural use but remains dependent on language skills [19]. Given the widespread use of the MoCA and its limitations, this study aimed to critically assess its application in a low-literacy Ugandan population, emphasizing the need for tailored adjustments [15, 20]. Given its global standardization and ease of administration, the MoCA was selected for this study to ensure comparability with the prior research while critically assessing its limitations and the need for scoring adjustments in low-literacy populations. The MoCA samples multiple cognitive domains relevant to early cognitive decline, but its total score has been shown to rely heavily on memory and orientation items, particularly in low-literacy populations [10, 21, 22].

In addition to the tools noted above, both the Mini-Mental State Examination (MMSE) and the Cognitive Screening Instrument for Dementia (CSID) are commonly used in LMIC settings. While both the Mini-Mental State Examination (MMSE) and the Cognitive Screening Instrument for Dementia (CSID) are widely used cognitive screening tools with relevance in LMIC contexts, this study focused on the Montreal Cognitive Assessment (MoCA) due to its superior sensitivity for detecting mild cognitive impairment (MCI) and its broader coverage of key cognitive domains, particularly executive function. Although the MMSE is the most universally administered cognitive screen, it has limited utility in early-stage cognitive decline and performs suboptimally in low-literacy and rural populations, where tasks involving visuospatial and executive processing are critical for early detection [23]. The MoCA has therefore gained increasing preference in global cognitive epidemiology, including in African settings.

For this study, the MoCA was linguistically and culturally adapted into Luganda using a forward–backward translation process and reviewed by local psychologists and linguists to ensure its semantic and conceptual accuracy. The adapted version was pilot-tested among older adults in Wakiso District to evaluate its clarity, feasibility, and appropriateness for a low-literacy population. While the MoCA has not been formally validated in Uganda, our adaptation was informed by successful regional efforts, such as the validated Swahili version used in rural Tanzania, which demonstrated feasibility in comparable populations [24]. In contrast, while the CSID is well-suited to low-education populations and has been validated in diverse settings, its longer administration time and greater training requirements made it less practical for our home-based, community screening approach.

The impact of education on cognitive test performance is well known, but there are limited data on the variations in MoCA scores by education level in sub-Saharan Africa. Past studies have typically used simple literate vs. illiterate classifications, neglecting finer educational distinctions [18]. The sex differences in MoCA scores are also underexplored in LMICs, despite the potential influences of sociocultural and educational disparities. Understanding these variations is crucial to creating fair and appropriate cognitive screening tools.

In sub-Saharan Africa, educational attainment varies widely due to historical, socioeconomic, and regional disparities, making it a critical modifier of cognitive screening performance. Although other sociodemographic variables—such as income, occupation, and rural residence—may also influence cognition, education is the most consistently documented and easily quantifiable determinant of performance in tools like the MoCA and the MMSE, particularly in low-literacy settings [25, 26]. This study prioritized the relationship between MoCA scores and educational attainment to understand how formal schooling contributes to cognitive performance in older Ugandan adults better. The educational categories used in this study (0, 1–3, 4–6, and 7–11 years) were selected to reflect meaningful stages within the Ugandan education system—ranging from no schooling, early to mid-primary education, to near-completion of primary school—and were informed both by theoretical significance and the empirical distribution in our sample. This stratification allows for a more contextually nuanced interpretation of the education-related differences in MoCA performance, building on prior findings that simple literate vs. illiterate groupings may obscure more subtle gradients in cognitive functioning [27].

This study quantified the impact of educational attainment on the MoCA performance among older adults in Uganda, where educational levels vary widely. We assessed the following: (i) the MoCA score differences across education levels (0, 1–3, 4–6, and 7–11 years), (ii) the suitability of MoCA’s standard education adjustment for this population, and (iii) the influence of sex on MoCA performance. Multiple linear regression was used to evaluate the effect of education on MoCA scores, adjusting for age and sex, and interactions were analyzed to determine whether the impact of education varied across demographic subgroups. Our findings highlight the cognitive screening challenges in LMICs and the need for localized MoCA scoring adjustments, directly benefiting clinicians, researchers, and policymakers working on dementia detection in low-resource settings.

## 2. Materials and methods

### 2.1. The study design and setting

This cross-sectional study was conducted between May and July 2023 in Wakiso District, Uganda, located within the Greater Kampala Metropolitan Area, which includes urban, suburban, and rural areas. The study period was chosen to align with stable community activity levels, ensure participant availability, and minimize seasonal variations in cognitive performance. This research focused on three sub-counties, Nansana (urban), Busukuma (rural), and Nakwero (rural), selected to capture the variations in educational attainment and cognitive performance across different socioeconomic backgrounds. These sub-counties have distinct educational and occupational profiles, with urban populations generally having greater access to formal education than rural populations do. Participants were selected through random sampling, ensuring a diverse representation of educational backgrounds to accurately assess the impact of education on MoCA scores.

### 2.2. The study population and recruitment

Participants were recruited from a previously established cohort of older adults in a 2018–2019 study on the burden of Alzheimer’s disease in Uganda. This parent study enrolled 500 elderly individuals from the Nansana and Busukuma sub-counties, providing a well-characterized participant base with documented cognitive and demographic profiles.

For this study, a random sampling approach was used to ensure an unbiased selection of participants from the cohort. Efforts were made to include both cognitively healthy individuals and those with prior cognitive concerns to ensure that the sample reflected a broader aging population. Although the recruitment was limited to two sub-counties, these areas represent a mix of urban and rural settings and share socioeconomic and demographic characteristics with other regions of Uganda, supporting this study’s broader relevance.

### 2.3. Inclusion and exclusion criteria

Participants were eligible for inclusion if they were aged ≥ 65 years and able to provide informed consent (or had a legally authorized representative (LAR)). To ensure consistency in the exposure to sociocultural and educational environments, only long-term residents were included. Participants were not excluded based on cognitive status to enable the full spectrum of MoCA performance to be assessed.

However, individuals were excluded if they had known neurological or medical conditions that could independently affect cognitive function in ways not reflective of typical age-related or Alzheimertype decline. These included

- HIV-associated neurocognitive disorder (HAND): Diagnosed based on national HIV treatment program records and confirmed through clinic documentation. HAND is known to produce distinct cognitive profiles, often affecting psychomotor speed, attention, and executive function disproportionately.
- Parkinson’s disease: Diagnosed clinically by a physician or neurologist; excluded due to its well-documented non-amnestic cognitive presentation and frequent medication-related cognitive fluctuations.
- Epilepsy: Excluded based on an active diagnosis or ongoing anti-epileptic treatment given potential effects on memory, processing speed, and attentional functioning.
- Other severe neurological conditions (e.g., stroke with significant residual deficits, traumatic brain injury) or psychiatric disorders that could have confounded the cognitive testing outcomes.

These exclusions were intended to reduce the diagnostic heterogeneity and ensure that the differences in MoCA performance could be more confidently attributed to educational attainment rather than disease-specific cognitive trajectories. Diagnoses were determined through a review of clinical records, medication histories, and structured informant interviews conducted by trained clinicians under the supervision of a psychiatrist and a neurologist.

### 2.4. The data collection procedures

The data were collected by a multidisciplinary research team, including trained clinical psychologists, nurses, and social workers, under the supervision of a psychiatrist. Before the data collection, all of the research assistants underwent two weeks of intensive training on MoCA administration, ethical research practices, and standardized data entry to ensure consistency in the cognitive assessments. To enhance the inter-rater reliability further, periodic quality control checks were conducted throughout this study.

Although the study team included clinicians and support staff, administration of the MoCA was conducted exclusively by trained clinical psychologists and experienced research assistants. All of the assessors received two weeks of standardized training in MoCA delivery and scoring, supervised by a senior neuropsychologist. The inter-rater reliability was assessed during pilot testing using 20 participants, with an intraclass correlation coefficient (ICC) of 0.87, reflecting excellent agreement. Weekly review meetings and direct supervision ensured continued fidelity to the standardized protocol throughout the study period.

All interviews were conducted in Luganda, the predominant local language, using a culturally adapted version of the MoCA. The adaptation process followed forward–backward translation protocols reviewed by a panel of psychologists and linguists to ensure semantic and conceptual equivalence. The adapted version was pilot-tested on 20 older adults before its full implementation to assess its clarity and feasibility.

To minimize test anxiety and literacy bias, the participants were assessed in familiar environments, such as their homes, under standardized conditions. The assessors ensured that testing was conducted in quiet, distraction-free areas, with the assessments scheduled at the optimal times of the day to reduce cognitive fatigue. Each session lasted 90–120 min, including cognitive testing and a structured sociodemographic questionnaire. To reduce participant fatigue, short breaks were offered upon request, and cognitive assessments were administered at the beginning of each session to capture the optimal performance levels.

### 2.5. Measures and variables

The primary measure was cognitive performance, assessed using the Montreal Cognitive Assessment (November 2004). For interpretive purposes, we applied commonly used MoCA cutoff scores from prior validation studies, defining ≥23 as normal cognition, 19–24 as mild cognitive impairment, and ≤18 as dementia [28]. These thresholds are widely referenced in the literature on global cognitive screening and were used in our descriptive analyses. However, to preserve analytical sensitivity, the MoCA scores were modeled as continuous outcomes in all of the primary statistical models. An adapted Luganda-translated version of the MoCA was used following forward–backward translation protocols to ensure accuracy. The independent variables were educational attainment, age, and sex. Education was categorized into four groups based on the highest number of years of schooling completed: 0 years (no formal education), 1–3 years, 4–6 years, and 7–11 years. Age and sex were included as covariates in the statistical models.

### 2.6. Bias mitigation strategies

Several measures were implemented to minimize bias and enhance the validity of the findings. Selection bias was addressed through a random sampling approach within the existing cohort, ensuring that all eligible participants had an equal chance of selection. A computer-generated randomization process was used to allocate participants into this study. Measurement bias was minimized through the standardized administration of the Montreal Cognitive Assessment (MoCA) by trained interviewers using scripted instructions and uniform scoring criteria to ensure consistency, while the inter-rater reliability was assessed before the data collection, requiring an agreement threshold of ≥85%. Expectation bias was mitigated by blinding the assessors to the participants’ medical histories during the cognitive assessments, and recall and response biases were reduced by cross-checking self-reported education levels with informant reports, resolving discrepancies through family members or historical documentation. Finally, potential confounding variables, such as age, sex, and literacy levels, were accounted for in the statistical analysis using multivariable regression models to isolate the effect of education on MoCA performance.

### 2.7. Sample size determination

The sample size was determined using a power analysis for multiple linear regression, considering three predictors: education, age, and sex. According to standard guidelines for regression analysis, the required sample size is calculated based on the number of predictors. With the three predictors, the estimated minimum required sample size to achieve a power of 80% at a significant level of 0.05 was calculated. The final study sample of 106 participants exceeded this threshold, ensuring sufficient power to detect statistically significant associations between the MoCA scores and the independent variables.

### 2.8. The statistical analysis

Descriptive statistics were used to summarize the sociodemographic characteristics of the study population. Continuous variables, such as age and MoCA scores, were presented as medians with interquartile ranges (IQRs) after confirming non-normality using the Shapiro–Wilk test. Categorical variables such as sex and education level were reported as frequencies and percentages.

Given the non-parametric nature of the data, Spearman’s correlation coefficient was used to assess the relationship between education and MoCA performance. Differences in MoCA scores across the four educational groups (0, 1–3, 4–6, and 7–11 years of schooling) were analyzed using the Kruskal–Wallis test, followed by Dunn’s post hoc test with Bonferroni correction to adjust for multiple comparisons.

Model diagnostics confirmed that the residuals met the normality assumptions, and robust standard errors were used to address heteroskedasticity. The covariate selection was based on the prior literature and Akaike Information Criterion (AIC) comparisons to ensure model parsimony. Interaction effects between education × sex and education × age were tested to explore whether the impact of education on MoCA varied across demographic subgroups. Effect sizes were reported using eta-squared for the Kruskal–Wallis tests and standardized beta coefficients for the regression models.

Cronbach’s alpha was computed to evaluate the internal consistency of the MoCA. Sensitivity analyses were performed to assess the impact of missing data by comparing the findings from complete-case analyses with those obtained using multiple imputation techniques (10 iterations, predictive mean matching) to ensure the robustness of the results.

### 2.9. Ethical considerations

This study was conducted in accordance with the Declaration of Helsinki and approved by the Ethics Committee of School of Health Sciences Research and Makerere University (MAKSHSREC-2022–395, approved on 11 April 2023) and the Uganda National Council of Science and Technology (HS3097ES, approved on 28 April 2023). Written informed consent was obtained from all of the participants. For those with cognitive impairments affecting their decision-making capacity, consent was provided by a legally authorized representative (LAR), typically a caregiver or next-of-kin. Decision-making capacity was assessed based on the MoCA scores and clinical judgments of trained assessors, ensuring that the participants were only assigned proxy consent if they lacked the ability to make informed decisions. Whenever possible, participants with mild cognitive impairments were asked for verbal or behavioral assent, and refusals were respected even if proxy consent had been obtained. The participants were informed of their voluntary participation and their right to withdraw at any time without any consequences. To safeguard confidentiality, all of the data were anonymized, securely stored, and made accessible only to authorized research personnel. The psychological risks associated with cognitive testing were minimized through a supportive interview approach, and participants who exhibited significant cognitive impairments were provided with information on follow-up care options. Ethical safeguards ensured that the assessments were conducted with dignity, respect, and participant well-being as priorities.

## 3. Results

### 3.1. The participants’ characteristics

A total of 106 participants were enrolled in this study, with a median age of 75 years (IQR: 70–82 years; range: 65–103 years). Their age was verified using national identification cards or family reports, minimizing potential errors in self-reported data. Most of the participants were female (78.3%, *n* = 83), while 21.7% (*n* = 23) were male. Their educational attainment varied significantly, with 47 participants (44.3%) having no formal education, 22 (20.8%) having completed 1–3 years of schooling, 18 (17.0%) having completed 4–6 years, and 19 (17.9%) having attained 7–11 years of education. The median number of years of formal education was three (IQR: 1–6; range: 0–11 years).

Their cognitive performance was assessed using the MoCA, with a median score of 11 (IQR: 7–16; range: 0–27). Exploratory classification using this median value revealed that 58 participants (54.7%) scored below this threshold. Among those scoring below 11, the majority (74%) had no formal education. This group also had higher rates of reported memory concerns reported by informants. MoCA scores were positively correlated with years of education (Spearman’s rho = 0.56, *p* < 0.001), indicating a moderate to strong association between education and cognitive performance. The Kruskal–Wallis tests revealed significant differences in the MoCA scores across educational groups (*p* < 0.001), with the post hoc Dunn’s tests confirming that individuals with no formal education scored significantly lower than those with ≥4 years of education (*p* < 0.01, Bonferroni-corrected). The demographic and baseline characteristics of the participants are summarized in Table 1.

### 3.2. The reliability of the Montreal Cognitive Assessment (MoCA) and internal consistency

The Cronbach’s alpha showed that the MoCA had acceptable reliability (α = 0.72). The item-wise reliability coefficients varied across the cognitive domains, with the highest item–test correlations observed for attention (0.62), naming (0.59), abstraction (0.54), and orientation (0.52). The lowest correlation was recorded for delayed recall (0.19), suggesting greater response variability in this domain. Table 2 presents the item-wise Cronbach’s alpha values.

### 3.3. The impact of education on the Montreal Cognitive Assessment (MoCA) scores

**Figure 1 •:**
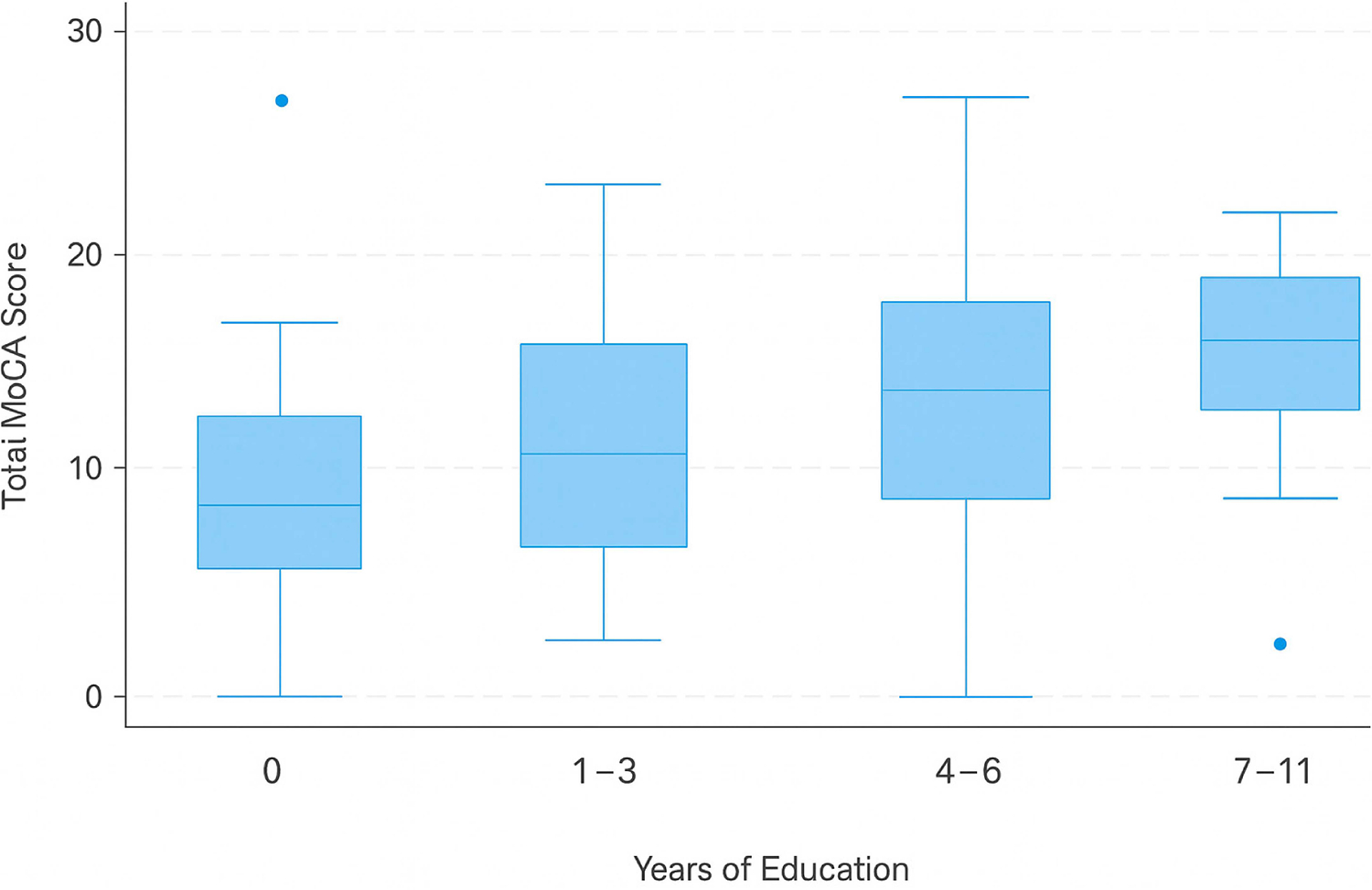
The variation in the MoCA scores with education. The blue circles on the boxplot denote outliers in the MoCA score distribution across the respective educational categories. The circle above the 0 years of education group signifies a participant with an unexpectedly high MoCA score, which is atypical, given the low level of education. Conversely, the circle below the 7–11 years of education group indicates a lower-than-anticipated MoCA score despite the relatively higher educational attainment.

The MoCA scores increased significantly with higher educational attainment (*p* < 0.001). The participants with no formal education had a median MoCA score of 8 (IQR: 6–10), whereas those with 1–3 years of schooling scored 12 (IQR: 10–14). A further increase was observed among individuals with 4–6 years of education (median: 15, IQR: 13–18), whereas the participants with 7–11 years of education had the highest median score of 17 (IQR: 15–21). These trends, as illustrated in Figure 1, underscore the impact of education on cognitive performance.

The Kruskal–Wallis test (H = 28.6, *p* < 0.001, η^2^ = 0.21) confirmed a statistically significant difference in the MoCA scores across education groups, with a large effect size (η^2^ ≥ 0.14, indicating strong effects). The post hoc Dunn’s tests with Bonferroni correction revealed significant differences in the MoCA scores between those with no formal education and all other groups (*p* < 0.01 for all comparisons). However, the difference between the 4–6-year and 7–11-year education groups was not statistically significant (*p* = 0.07, Cohen’s d = 0.38), suggesting that the most substantial cognitive benefits were associated with obtaining at least some formal education but that these improvements plateaued beyond primary schooling.

To ensure that these findings were not confounded by age or sex differences, an additional multivariate regression analysis was conducted adjusting for these variables. The results remained robust, confirming that education was a significant predictor of MoCA scores, independent of age and sex effects.

### 3.4. The sensitivity analysis using dichotomized educational attainment

**Figure 2 •:**
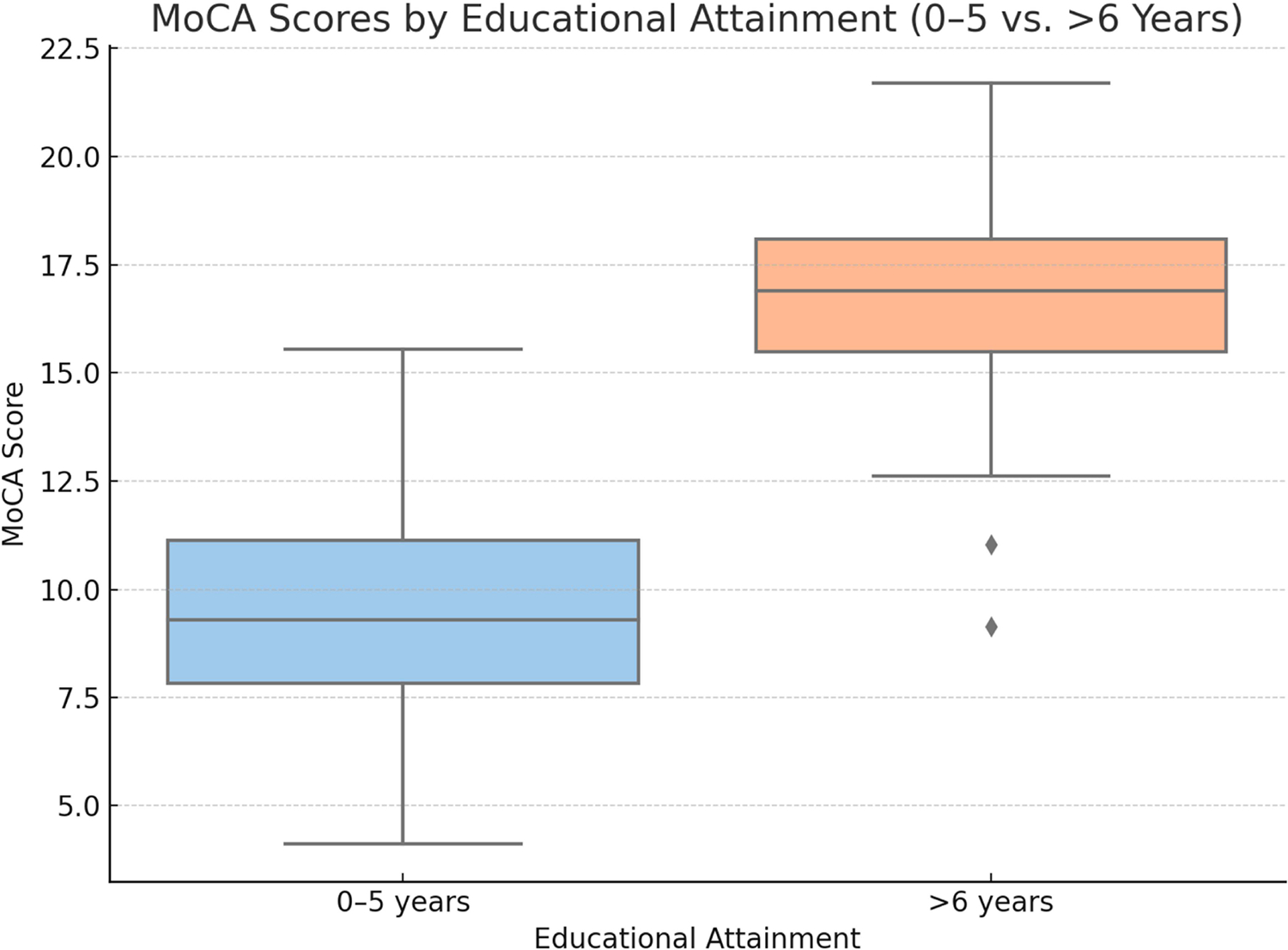
MoCA scores by educational attainment (0–5 vs. >6 years). The boxes represent the interquartile range (IQR), with the horizontal line indicating the median. Whiskers extend to the most extreme values within 1.5 times the IQR. Diamonds represent outliers, defined as values beyond 1.5 times the IQR from the first or third quartile.

To evaluate the robustness of our findings, a sensitivity analysis was conducted by dichotomizing educational attainment into two groups: 0–5 years and >6 years. Participants with more than 6 years of education had significantly higher MoCA scores (median: 17; IQR: 15–21) compared to the scores in those with 0–5 years (median: 10; IQR: 7–13; *p* < 0.001, Mann–Whitney U test). A multivariable regression model using this binary education variable, adjusting for age and sex, confirmed that education remained a significant independent predictor of MoCA performance (β = 4.25, 95% CI: 2.67–5.84, *p* < 0.001), with a model fit similar to that in the original analysis (Adjusted R^2^ = 0.41). These findings support the consistency and robustness of the association between educational attainment and cognitive performance across different grouping strategies. The distribution of the MoCA scores by dichotomized education level is illustrated in Figure 2.

### 3.5. Sex disparities in Montreal Cognitive Assessment (MoCA) performance

On average, males had a higher mean MoCA score (13.75, SD: 5.6) than that in females (12.0, SD: 5.0), with a statistically significant difference (t (104) = 2.17, *p* = 0.032, Cohen’s d = 0.35), indicating a small to moderate effect size. The normality of the MoCA scores was confirmed using the Shapiro–Wilk test (*p* > 0.05), supporting the use of a parametric t-test.

A linear regression model (F (2, 103) = 18.43, *p* < 0.001, R^2^ = 0.14) revealed that both age and sex significantly predicted MoCA scores. Older participants had lower MoCA scores (β = −0.15, t = −6.32, *p* < 0.001), while males scored significantly higher than females (β = 2.36, t = 3.12, *p* = 0.002). However, the age × sex interaction (*p* = 0.59) was not significant, indicating that the impact of aging on MoCA performance was similar in both sexes.

**Figure 3 •:**
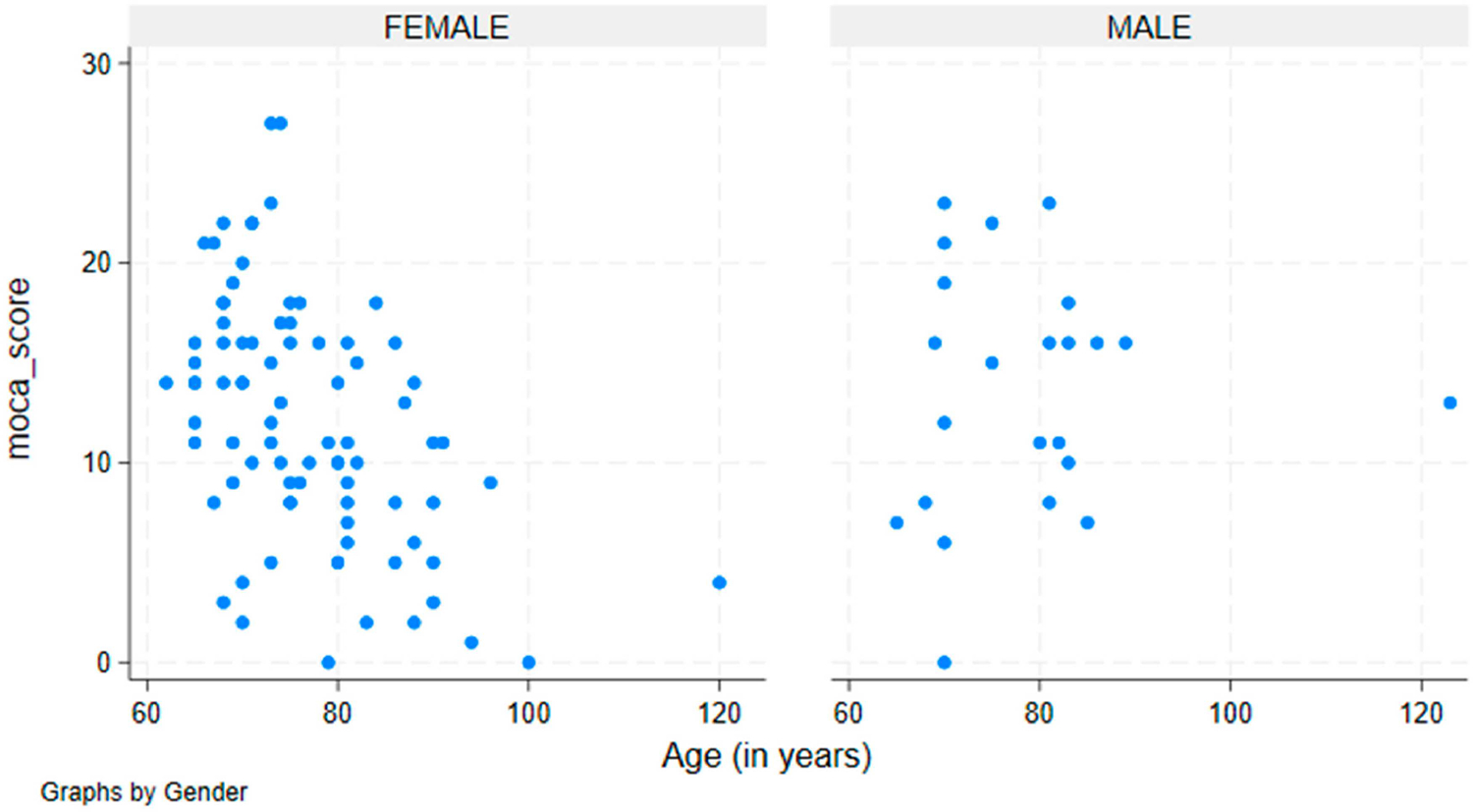
Average MoCA scores stratified by sex. Each blue circle represents an individual participant. The plot illustrates the distribution of cognitive scores (MoCA) across different ages among males and females.

Despite the statistical significance of these findings, the regression model explained only 14% of the variance in the MoCA scores, suggesting that additional factors such as education level, literacy, and socioeconomic status may be key determinants of cognitive performance. These findings are illustrated in Figure 3.

### 3.6. The multivariable regression analysis

A multiple linear regression model was fitted to assess the independent effect of education on the MoCA scores after adjusting for age and sex. Education emerged as the strongest predictor of MoCA performance (β = 1.73, 95% CI: 1.22–2.24, *p* < 0.001, Cohen’s f^2^ = 0.32), indicating a large effect size relative to that of the other predictors. Age remained negatively associated with MoCA scores (β = −0.13, 95% CI: −0.19 to −0.07, *p* < 0.001), while male sex was positively associated (β = 1.89, 95% CI: 0.56–3.22, *p* = 0.005).

Interaction terms were tested to explore whether the relationship between education and MoCA scores differed by age or sex; however, education × sex (*p* = 0.11) and education × age (*p* = 0.27) were significant, suggesting that the effect of education on cognitive performance was stable across different demographic groups.

The final regression model explained 42.7% of the variance in the MoCA scores (adjusted R^2^ = 0.43, *p* < 0.001). Model diagnostics confirmed that assumptions of normality, multicollinearity (VIF < 2.0), and homoscedasticity were met, supporting the validity of the linear model.

## 4. Discussion

Our study quantified the impact of educational attainment on performance in the Montreal Cognitive Assessment (MoCA) among older adults in Uganda, revealing significant disparities in cognitive scores across educational levels. These findings underscore the critical role of formal schooling in the outcomes of cognitive assessment and highlight the potential for misclassification in populations with limited educational exposure. This discussion contextualizes our results within the broader framework of cognitive screening in low- and middle-income countries (LMICs); examines the implications for dementia diagnoses; and offers recommendations for future research and clinical practice.

Consistent with previous studies, our analysis demonstrated a robust positive correlation between years of education and MoCA scores (Spearman’s rho = 0.56, *p* < 0.001), emphasizing the substantial influence of formal education on cognitive test performance [29, 30]. Participants with no formal education exhibited significantly lower MoCA scores than the scores in those with minimal schooling, with the most pronounced differences observed between individuals with no education and those with at least four years of schooling. These findings align with research from other LMICs, including India and China, where MoCA scores have been shown to be highly dependent on literacy and educational background [13, 14].

The strong association between education and MoCA performance observed in our study aligns with prior research from sub-Saharan Africa and other LMICs, where formal schooling has consistently emerged as a key determinant of cognitive screening outcomes. While many previous studies used broad literate/illiterate classifications, this approach can obscure the more nuanced effects of partial or primary-level education on test performance [27]. Our use of four educational categories—reflecting the transitions within Uganda’s school system—allowed for more granular insight into how specific thresholds of schooling related to the cognitive test scores. This approach builds on evidence from regional validations, such as the Kiswahili MoCA study in rural Tanzania, which similarly emphasized the need for culturally and educationally tailored screening strategies [26]. These findings underscore the importance of moving beyond binary classifications and adopting context-sensitive stratification schemes that reflect the diversity of educational attainment in African populations of older adults better.

Our results call into question the validity of the standard education adjustment in the MoCA (+1 point for individuals with ≤12 years of education) in the Ugandan context. The marked disparities in MoCA scores between educational groups suggest that a single-point correction does not sufficiently account for differences in familiarity with cognitive tests and literacy-related cognitive skills. Similar concerns have been raised in studies from Brazil and China, where stratified or modified MoCA scoring approaches have been recommended to enhance the diagnostic accuracy in low-literacy populations. Additionally, our findings indicate a plateau effect in cognitive scores beyond primary education, suggesting that while early schooling provides measurable cognitive benefits, additional years of education yield diminishing returns in terms of improvements in MoCA performance [31].

Given the high prevalence of individuals with little or no formal education in sub-Saharan Africa, these findings have critical implications for screening for dementia. Despite its inherent educational bias, reliance on the MoCA in LMICs raises concerns regarding the validity of cognitive assessments in older adults with minimal schooling. Overestimation of cognitive impairment in illiterate or low-literacy individuals may result in unnecessary anxiety, misclassification, and inappropriate clinical referrals, whereas underestimation in individuals with higher education may lead to delayed dementia diagnoses.

Alternative cognitive screening tools, such as the Rowland Universal Dementia Assessment Scale (RUDAS) and the IDEA cognitive screen, which reduce the dependence on language and formal education, have shown promise in LMIC settings [32–34]. However, the MoCA remains the most widely used cognitive assessment tool in both clinical and research contexts, necessitating further adaptations to enhance its accuracy across educationally diverse populations. Our findings reinforce the need for region-specific MoCA modifications, including stratified cutoffs or localized scoring adjustments that reflect educational variability.

Building on the initial hypothesis that sex may influence MoCA performance, our findings confirm significant disparities, possibly reflecting broader educational and sociocultural differences, with males scoring higher than females (*p* = 0.032). Although the effect size was small to moderate (Cohen’s d = 0.35), this finding suggests that sociocultural factors may have influenced cognitive test performance. In many LMICs, historical sex disparities in access to education may contribute to differential cognitive test scores, as women, particularly in older cohorts, have often been less likely to have received formal schooling [35, 36]. Similar sex-related disparities in cognitive screening outcomes have been documented in other LMIC studies, underscoring the need for further investigation of the interplay between education, sex, and cognitive aging.

Despite the observed sex effect, our regression analysis confirmed that education remained the strongest predictor of MoCA performance independent of sex and age. This suggests that addressing educational disparities in cognitive screening should be prioritized over sex-specific scoring adjustments to improve the accuracy and equity of dementia diagnoses in LMIC settings.

Building on this, an additional consideration is the use of locally derived thresholds for interpreting the MoCA scores in low-literacy populations. The use of a population-specific median MoCA score as a reference point for classifying cognitive impairment is a pragmatic approach in LMIC settings, particularly in communities with low literacy. Our median MoCA score of 11 aligns with similar studies in rural sub-Saharan Africa and underscores the limitations of relying on the thresholds from high-income countries. While not intended to replace a clinical diagnosis, this data-driven classification could serve as a contextual screening benchmark until formal validation studies establish education-adjusted local norms.

An unexpected observation in our cohort was that male participants demonstrated significantly higher MoCA scores than those in their female counterparts. This contrasts with many population-based studies where either minimal sex differences have been reported or females have slightly outperformed males in cognitive screening, particularly in memory-related tasks [37]. In our study, however, the men had notably higher levels of formal education than the women, which may have partly explained this difference. Furthermore, the smaller number of male participants may have introduced a selection bias, with more educated or cognitively intact men being more likely to participate. These factors may have contributed to the observed male advantage and should be interpreted cautiously.

Although years of formal education were carefully recorded and stratified in our analysis, we acknowledge that literacy was not directly measured. In sub-Saharan Africa, years of schooling may not uniformly reflect actual literacy skills due to differences in school quality, interruptions in attendance, and limited access to reading materials [37, 38]. Consequently, individuals with similar education levels may have had vastly different reading and comprehension abilities, which could have influenced their MoCA performance, particularly in tasks requiring language processing or abstraction. This limitation introduces potential unmeasured variability into our findings, and future studies should consider including direct assessments of literacy to contextualize the cognitive screening results better.

## 5. Conclusions

This study highlights the significant impact of educational attainment on the performance in the MoCA among older Ugandans, emphasizing the need for localized cognitive screening adaptations in LMICs. Future research should focus on developing and validating region-specific MoCA adjustments for low-literacy populations. Policymakers and clinicians should consider alternative cognitive screening tools, such as the RUDAS and the IDEA screen, to minimize literacy bias. Implementing education-sensitive screening protocols and training healthcare professionals to interpret MoCA scores appropriately would improve the accuracy of dementia diagnosis. Collaborative efforts among researchers, policymakers, and community health workers are essential for refining cognitive assessment strategies for diverse populations.

## Figures and Tables

**Table 1 •:** Demographic characteristics and cognitive measures of study participants.

Factor	Level	Frequency (%)/value	Ranges of values
Number of years in education	Median (IQR)	3(1,6)	0 to 11
Age	Median (IQR)	75(70,82)	62 to 123
Sex	Female	83(78.3%)	
	Male	23(21.7%)	
MoCA score	Average	12.3	0 to 27

**Table 2 •:** Cronbach’s alpha values for the MoCA items.

Item	Observations	Sign	Item test correlation	Alpha
Visuospatial score	106	+	0.45	0.68
Naming score	106	+	0.59	0.66
Attention score	106	+	0.62	0.63
Language score	106	+	0.42	0.70
Abstraction score	106	+	0.54	0.68
Delayed recall score	106	+	0.19	0.76
Orientation score	106	+	0.52	0.67
Test scale	106	+		0.72

## Data Availability

The data supporting the findings of this publication can be made available upon request.
